# Effects of emotionally oriented parental interventions: a systematic review and meta-analysis

**DOI:** 10.3389/fpsyg.2023.1159892

**Published:** 2023-07-13

**Authors:** Rune Zahl-Olsen, Linda Severinsen, Jan Reidar Stiegler, Carina Ribe Fernee, Indra Simhan, Sondre Sverd Rekdal, Thomas Bjerregaard Bertelsen

**Affiliations:** ^1^Department of Child and Adolescent Mental Health, Sorlandet Hospital, Kristiansand, Norway; ^2^Department of Psychology, University of Oslo, Oslo, Norway; ^3^Institute for Psychological Counselling, Bergen, Norway; ^4^Department of Sport Science and Physical Education, University of Agder, Kristiansand, Norway

**Keywords:** emotion-focused, parental intervention, emotion socialization, emotionally oriented, child outcomes, parent outcomes

## Abstract

**Objective:**

This systematic review and meta-analysis investigates the effects of emotionally oriented parental interventions.

**Background:**

Several emotionally oriented parental interventions have been developed during the last decade. Some of these have gained popularity and spread across several continents. The literature is growing and consists of qualitative studies; intervention only, quasi-experimental, case-control studies; and randomized controlled trials. They indicate effects for parents and children. However, no systematic review or meta-analysis has, to our knowledge, summarized the results.

**Method:**

Using several search engines, we located 8,272 studies. After abstract and full-text screening, 33 studies were assessed for bias and included in the study. Outcomes for parents and children were extracted and combined into three constructs for parents and two for children. Meta-analyses were conducted for each construct to estimate the effect of the interventions using a robust Bayes meta-analysis.

**Results:**

The results indicate the presence of a small to medium effect on parents' mental health, behavior, and use of emotionally oriented parenting, as well as on children's internalizing and externalizing difficulties. Most participants were recruited from the general population, and clinical settings were rare. The results show little evidence of publication bias.

**Conclusion:**

There is evidence of a small to medium effect of emotionally oriented interventions on parents and children.

**Systematic review registration:**

https://osf.io/un3q4/.

## 1. Introduction

The extent of mental health difficulties in children is substantial (Merikangas et al., 2010; Catalano and Kellogg, 2020; Danielson et al., 2021), rising (Collishaw et al., 2004; Collishaw, 2015), and potentially higher than previously estimated (Deighton et al., 2019). International prevalence studies estimate that about 13–17% of children and adolescents experience mental illness (Barkmann and Schulte-Markwort, 2010; Polanczyk et al., 2015; Kovess-Masfety et al., 2016). Mental health difficulties in childhood are predictors of several negative trajectories leading to further mental health difficulties (Otto et al., 2020; Winsper et al., 2020; Arslan et al., 2021) and impaired quality of life (Barican et al., 2022; Piao et al., 2022).

The last two decades have shown a promising increase in interventions that target children's mental health difficulties (Steele et al., 2020). However, a large proportion of children with mental health difficulties are not receiving appropriate care (Halfon et al., 2012; Whitney and Peterson, 2019). In addition, a considerable proportion of those receiving care do not show sufficient improvement (Eyberg et al., 2008).

Although innate factors within the child and social factors surrounding the child contribute greatly to the development of children's mental health difficulties (Humphrey and Wigelsworth, 2012; Felitti et al., 2019), it is also assumed that the quality of the interaction between children and their attachment figures is essential to children's development and wellbeing. Parents' mental health and parental skills are assumed to play key roles in the trajectories of children's mental health development. Parental mental health issues, such as depression, are considered a significant risk factor for mental health issues in children (Lovejoy et al., 2000; Hannigan et al., 2018). Parental mental health appears to act as a mediator in a number of favorable mental health outcomes in children, including improved emotional, psychological, and developmental functioning (Willner et al., 2016; Rayce et al., 2020). Studies suggest that aiding children through their parents is a promising and effective approach to reduce mental health issues in children (Lundahl et al., 2006; Comer et al., 2013; Buchanan-Pascall et al., 2018). Another suggested strategy for alleviating mental health difficulties in children is to develop and implement strategies aimed at strengthening parents' mental health and parental skills (National Academies of Sciences, 2019).

One increasingly recognized mechanism is parents' ability to help children understand, regulate, and deal with their emotions in healthy ways (Gottman, 1997; Morris et al., 2017b). Children of parents who respond in an emotionally healthy manner (i.e., helping their child to acknowledge, understand, and regulate their emotions) seem to have fewer symptoms of mental health difficulties (Perry et al., 2020; Cooke et al., 2022). Conversely, unhealthy ways of dealing with children's emotions (i.e., neglectful, harsh, or coercive parenting styles, with little acceptance of experiencing or expressing a range of emotions) are associated with poorer mental health in children (Compas et al., 2017), adolescents (Schäfer et al., 2017), and adults (Cloitre et al., 2019). Furthermore, parents with unhealthy emotion regulation strategies are prone to negatively impact their children's emotion regulation strategies (Morris et al., 2007), while parents with healthy emotion regulation strategies seem to provide their children with a “psychological immune system,” helping their children better cope with life challenges (Morris et al., 2017a).

In recent years, several programs have been developed to strengthen the emotional interplay between parents and their children (see, for instance, Havighurst et al., 2004; Thomas et al., 2017; Weiss et al., 2018; Burgdorf et al., 2019). These programs have in common that they focus on parents' abilities to recognize, verbalize, and display an accepting attitude toward their children's emotional state. They do not directly involve the child but are, rather, designed to effect observable changes in parents. The concept behind such programs is that, because parents play such an important part in a child's everyday life, parental programs will have a greater effect on the child's mental health than weekly individual child therapy sessions (Dolhanty et al., 2023). Given the effect of parenting on children's mental health, one would assume a sequence of changes that begins with the parent, leading to a change in the interaction between the parent and child, which ultimately leads to a change in the child themselves. The change in the child may be delayed rather than immediate. It is therefore of relevance for parental programs to not only measure the child's outcomes but also the change in the parent-child interaction. Studies have shown a promising effect on these types of interventions on parental self-efficacy, as well as children's internalizing and externalizing difficulties (e.g., Ansar et al., 2022). Although these programs are promising ways of intervening in children's mental health issues, more research is needed to draw conclusions regarding their effectiveness (England-Mason and Gonzalez, 2020). Recent reviews include Havighurst et al.'s (2020) overview of emotion-focused parenting intervention studies published during an 18-month period, which identified 50 publications during 2019–2020. Studies varied in that some included community samples, whereas others were conducted with clinical populations, demonstrating a promising evidence base for emotion-focused parenting interventions. England-Mason et al. (2023) carried out a systematic review and meta-analysis limited to randomized controlled trials of emotion socialization parenting interventions targeting emotional competence in young children. Twenty-six studies reported data from 15 individual trials, whereby the authors concluded that these interventions are effective for improving emotion socialization parenting practices and child emotional competence. Still, methodologically rigorous trials are required to solidify current evidence and more insights into moderating factors are requested (England-Mason et al., 2023). The current paper extends these reviews by conducting a systematic review and meta-analysis of a larger proportion of studies including both randomized controlled trials and observational studies covering the entire evidence base of emotionally oriented parental interventions.

### 1.1. Current study

Emotionally oriented interventions for parents are manualized, intensive, skills-oriented programs designed to enhance parents' capacity to coach or guide their children to better recognize, understand, and express their emotions in healthy ways. The intervention dominating the literature is the Tuning into Kids program, developed by Havighurst et al. (2004), which has been adapted to serve various age groups and settings. Another parental intervention is Emotion-Focused Skills Training (EFST), which was first developed under the name Emotion-Focused Family Therapy (EFFT). Emotion-Focused Family Therapy was originally designed for families in which children were suffering from eating disorders (Dolhanty and Lafrance, 2019). The program has since been adapted for a broader range of mental health difficulties. Even though the concepts used to describe the interventions differ (e.g., emotion-focused, emotion socialization, emotional competence, and emotion regulation), they all seem to be based on the same core principles as Emotion-Focused Therapy for couples and individuals (Greenberg and Goldman, 2019). A central assumption of this model is that a person's affective system is developed and matured in interaction with significant others. The programs are targeted at helping parents provide their children with emotional experiences that enable healthy adjustments to their environment. Due to an increased interest in these parental programs, there is a need to summarize their effects on mental health among children and their parents. Our research question concerns whether there is evidence of an effect of emotionally oriented parental programs on children's and parents' mental health, as well as parental behavior toward the child. This study is a systematic review and meta-analysis of current evidence.

## 2. Methods

This study was pre-registered at an open science platform (osf.io). We used the Preferred Reporting Items for Systematic Reviews and Meta-Analyses (PRISMA) and the ASreview (van de Schoot et al., 2021) software to screen article titles and abstracts. The R software (R Core Team, 2017) with the RoBMA package (Bartoš and Maier, 2020) was used to conduct meta-analyses.

We included studies in which parents participated in an emotionally oriented program with the aim of improving their children's mental health. We included studies of any type of mental health intervention that was identified as emotionally oriented and in which the children or youth were below the age of 18. In addition, the intervention had to be identified as a parental intervention aiming at helping children indirectly, through their parents. Focusing on parental interventions only, we excluded studies in which children participated. We included observational and randomized controlled trials of any design that described the effectiveness of interventions (i.e., at least pre- and post-intervention measures and/or change in outcomes). We also included studies that investigated the process of change.

The primary outcomes are children's externalizing behavior and internalizing symptoms, which can be assessed in terms of diagnostic status, symptoms, or functional impairment via self-/parent reports, observation, or clinical interviews. Secondary outcomes, when reported, are parents' mental health, parent behavior, and the level of emotionally oriented parenting.

We searched in the Ovid Medline, Ovid Embase, Ovid PsycInfo, CINAHL, Cochrane Library, Epistemonikos, and PubMed databases with the assistance of librarians. The main search terms were CHILD, ADOLESCENT, PARENT, FAMILY, EMOTION-FOCUSED THERAPY, EMOTION REGULATION, and EMOTION SOCIALIZATION. These terms were adjusted according to the database searched, and keywords were included. We also conducted a backwards (cited) and forwards (citing) citation analysis by hand, in addition to analysis of citation network in CoCites and Connected papers. We included peer-reviewed, full-text publications. We excluded studies that were only available as abstracts and studies published in other languages than English, Norwegian, Swedish, and Danish.

**Figure 1:**
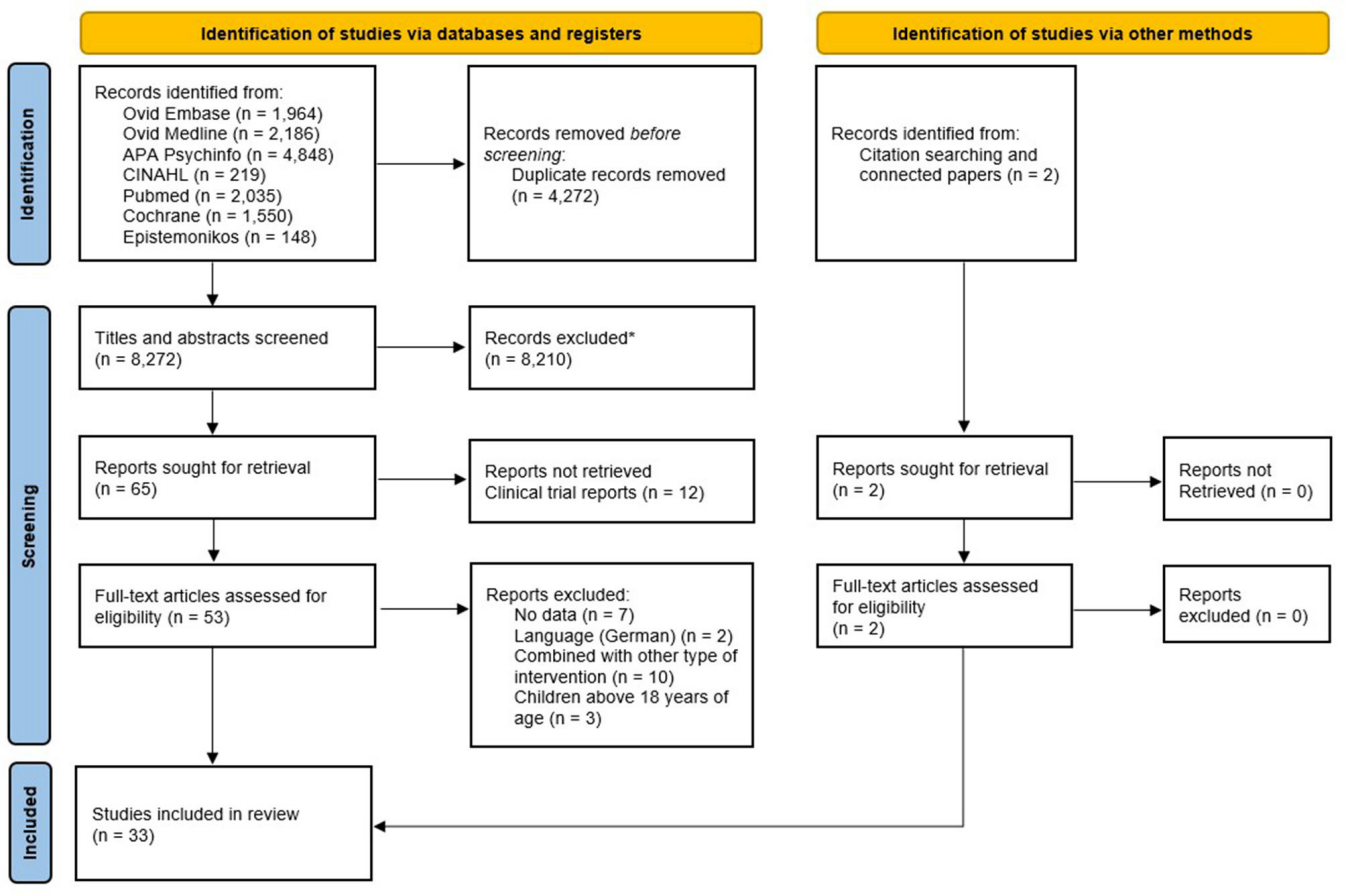
PRISMA flow diagram.

Four authors (RZO, LS, IS, and CRF) independently screened the titles and abstracts for inclusion against the inclusion criteria using ASreview software (van de Schoot et al., 2021). The same four authors read the full-text articles and determined the final inclusions collaboratively. Any disagreements were resolved via discussion. The selection process is recorded in the PRISMA flow diagram, as depicted in Figure 1, and a bibliography of all included studies and a list of excluded full-text studies are available in Supplementary material.

### 2.1. Data extraction

We extracted the following data from the included studies:

1. Methods: study authors, location, study design, duration of follow-up.
2. Participants: N, age (mean/median, range/SD), type of mental health disorder, sex.
3. Intervention: group/individual.
4. Comparators: time between pre-treatment and assessment.
5. Outcomes: four outcome types were specified for the children, which were mental health symptoms (continuous rating-scales), diagnostic status (dichotomy), functional impairment (continuous rating-scales), and wellbeing (continuous rating-scales). Parental mental health and parental functioning were measured using continuous rating-scales.

A summary of the extracted data for each included study is found in Table 1.

**Table 1:** General data derived from and bias assessment for the included studies.

	**References**	**Inter-vention**	**Country**	**Design**	**RCT**	**Child age**	**Session length**	**Type of delivery**	**N-for analysis**	**Parent measures**	**Child measures**	**Longest follow-up**	**Bias**
1	Aghaie Meybodi et al. (2017)	TIK	Iran	1 (a)	0	3–6	6 × 2 h + 2 booster	Group/weekly	25 (1)	1, 4	1, 2	3	Serious
2	Aghaie Meybodi et al. (2019)	TIK	Iran	1 (a)	1	3–5	6 × 2 h + 2 booster	Group/weekly	25 (1)	2, 3	1, 2, 5	3	Serious
3	Ansar et al. (2021)	EFST	Norway	0	0	6–13	2 days + 6h	Group + one-to-one	14				
4	Ansar et al. (2022)	EFST	Norway	1	1	6–13	3 days + 6h	Group + one-to-one	120, 116		x	12	Moderate
5	Bjørk et al. (2022)	TIK	Norway	1 (b)	0	5–6	6 × 2 h	Group/weekly	20 (2)	3	x	6	Moderate
6	Bølstad et al. (2021)	TIK	Norway	2 (b)	1	5–6	6 × 2 h	Group/weekly	21 (2)	1	1, x	Post (8)	Moderate
7	Chan et al. (2021)	TIK	China	1	1	3–6	6 × 2 h	Group/weekly	54	3, x	5	6	Moderate
8	Edrissi et al. (2019)	TIK	Iran	1	1	4–6	6 × 2 h	Group/weekly	30		x	1.5	Moderate
9	Foroughe et al. (2019)	EFFT	Canada	1	0	2–19	2 days	Group	90	x	3	4	Moderate
10	Havighurst et al. (2004)	TIK	Australia	1	0	4–5	6 × 2 h	Group/weekly	47	3, 4, x	1, 3, x	3	Moderate
11	Havighurst et al. (2009)	TIK	Australia	1 (c)	1	4–5	6 × 2 h	Group/weekly	107 (3)	1, 2, 4	1	Post (2.5)	Moderate
12	Havighurst et al. (2010)	TIK	Australia	2 (c)	1	4–5	6 × 2 h	Group/weekly	106 (3)	1, 2, 7	1, x	6	Serious
13	Havighurst et al. (2013)	TIK	Australia	1	1	4–5	6 × 2 h	Group/weekly	31	1, 2, 7	1, x	6	Moderate
14	Havighurst et al. (2015)	TINT	Australia	1 (d)	1	10–13	6 × 2 h	Group/weekly	121 (4)	2, 6, x		Post (10)	Moderate
15	Havighurst et al. (2019)	TIK	Australia	1	1	4–5	6 × 2 h	Group/weekly	87	1, 3, 5, x	3	Post (10)	Moderate
16	Havighurst et al. (2021)	TF-TIK	Australia	1	0	3–15	10 × 2 h	Group/weekly	77	1, x	1, 5	Post (Imm)	Serious
17	Havighurst et al. (2022)	TOTS	Australia	1	1	1.5–3	6 × 2 h	Group/weekly	163	1, 2, 3	x	12	Moderate
18	Kehoe et al. (2014)	TINT	Australia	1 (d)	1	10–13	6 × 2 h	Group/weekly	121 (4)	2, 4, 6	4	Post (10)	Moderate
19	Kehoe et al. (2015)	TINT	Australia	2 (d)	1	10–13	6 × 2 h	Group/weekly	121 (4)	2, 4, 6	2, 4, x	Post (10)	Serious
20	Kehoe et al. (2020)	TINT	Australia	3 (d)	1	10–13	6 × 2 h	Group/weekly	121 (4)	2, 4, 6	2, 4, x	Post (10)	Serious
21	Lambie et al. (2020)	MFE	England	0	0	2–5	4 × 1 h	Group vs. one-to-one	11				
22	Lauw et al. (2014)	TOTS	Australia	1	0	1.5–3	6 × 2 h	Group/weekly	34	1, 3, 7	x	Post (Imm)	Moderate
23	Leung et al. (2020)	HPRCP	China	1	1	3–7	8 × 2 h	Group/weekly	57	x	1	2	Moderate
24	Mastromanno et al. (2021b)	TIK	Australia	1	0	4–10	8 × 1 h	One-to-one/11 weeks	3 (5)	2, 3, x	1, 4	6	Moderate
25	Mastromanno et al. (2021a)	TIK	Australia	1	1	4–10	8 × 1 h	One-to-one/11 weeks	51 (5)	3, x	1, 4	6	Moderate
26	Pezeshki (2020)	ECPP	Iran	1	1	3–5	7 × 2 h + 1 booster	Group/weekly	15	1, 2, x	2	3	Moderate
27	Qiu and Shum (2022)	TIK	China	1	1	3–6	6 × 2 h	Group/weekly	42, 39	1, 3, 4, x	3, x	Post (Imm)	Moderate
28	Rolock et al. (2021)	TINT	USA	1	1	10–13	7 × 2 h	Group/weekly	327	x		8	Moderate
29	Shortt et al. (2014)	ETCYC	USA	1	0	4–12	15 × 2 h	Group/8 weeks	29	2, x		6	Moderate
30	Wilhelmsen-Langeland et al. (2020)	EFFT	Norway	1	0	6–12	2 days	Group	23	5, x	2	3	Serious
31	Wilson et al. (2012)	TIK	Australia	1	0	4–5	6 × 2 h	Group/weekly	62	1, 4, x	x	7	Serious
32	Wilson et al. (2014)	TIK	Australia	1	0	3–6	7 × 2 h	Group/weekly	43	1, 3, 5, x	3	Post (Imm)	Moderate
33	Wilson et al. (2016)	TIK	Australia	1	1	3–6	7 × 2 h	Group/weekly	87	1, 3, 5, x	3	Post (Imm)	Serious

Regarding N: Because we only include participants offered the active treatment the N here is lower than mentioned in the studies. Some studies use the same sample, this is indicated by a number within brackets. When more than one N occur it indicate that the study has several samples offered the intervention.

Regarding intervention: TIK = all variations of tuning into kids, EFST = all variations of emotion-focused skills training, EFFT = emotion-focused family therapy, ECPP = emotion coaching parenting program, MFE = my first emotions, ETCYC = emotions: taking care of yourself and your child when you go home, HPRCP = happy parenting: round-the-clock parenting program.

Regarding parent measures: 1 = PESQ, 2 = DERS, 3 = CNES, 4 = GHQ, 5 = PSOC, 6 = EAC, 7 = Video, x = other measure.

Regarding child measures: 1 = ECBI, 2 = CBCL, 3 = SDQ, 4 = SCAS, 5 = ERC, x = other measure.

Regarding design: 1 indicate that the study has a quantitative design. 0 indicate a qualitative design. The parenthesis indicate that the studies are based on the same sample.

Regarding intervention: TOTS, TF-TIK, and TINT are variants of TIK.

Regarding the longest follow-up, for post-measures, the information within brackets indicates when the post-data was collected. Imm indicates data collection immediately after intervention, and numbers indicate months after baseline data.

### 2.2. Assessment of risk of bias in included studies

We used the Robins-I tool to assess the risk of bias in all studies (Sterne et al., 2016) because our research question concerns whether there is an effect at all, not whether there is an effect as compared to another intervention. Four authors (RZO, LS, IS, and CRF) assessed the risk of bias. Any disagreements were resolved via discussion, and the assessment is presented in Table 1. According to Sterne et al. (2016), if a study is assessed as having a serious risk of bias in one domain, the total assessment of bias for the study should be concluded as serious or worse, even if the risk of bias is assessed as lower in other domains. However, to differentiate the included studies, we did not include measurement bias in our final assessment. The reason for this choice was that all studies were assessed as having serious risk of biased outcomes within this domain because blinding is not possible within our field of research and the studies used self-assessment measures.

### 2.3. Measurement of effect and data synthesis

For dichotomous outcomes, we use the odds ratio and the standardized mean difference $\left(Cohens\;d\;=\;\frac{\mu_{2}-\mu_{1}}{\sigma }\right)$, where σ is the pooled standard deviation for continuous outcomes (Cohen, 1988).

### 2.4. Unit of analysis

Our intention was to conduct the analysis on original data derived from all included studies. However, only five of 15 corresponding authors responded to our data request. Considering the fact that this only provided us with data on five of the 32 included studies, we instead opted for an analysis using sample-level summary statistics, as reported in the selected study reports. If several outcomes measuring the same construct were presented within a paper, these were then combined following the procedures outlined in Harrer et al. (2021). When several studies drew on the same underlying dataset, the effect-measures were combined, again following recommendations on the part of Harrer et al. (2021).

### 2.5. Measures

The main aim of a meta-analysis is to determine how a construct changes based on several studies. Because the measures used in the included studies varied, we were unable to select a single measure for use in our meta-analysis. Thus, we followed the procedures outlined in Harrer et al. (2021), combining several outcomes measuring the same constructs within and across studies. As authors, we represent several professions, specifically psychiatry, psychology, and couples and family therapy, and have expertise in emotion-focused treatment, family therapy, narrative therapy, outdoor therapy, and cognitive behavioral therapy. We discussed what constructs would be meaningful for clinicians and researchers within psychotherapy in general, as well as those that are well known in the emotionally oriented literature, and arrived at two constructs for the children's measures and three constructs for the parents' measures.

Twenty-nine studies included measures of change for the children's internalizing or externalizing difficulties. A total of 19 measures were used. Of these, eleven (58%) of the measures were not used in more than one study, whereas three measures (16%) were applied in two studies. The most common measures were the Eyberg Child Behavior Inventory (ECBI; k = 11), the Child Behavioral Checklist (CBCL; k = 6), the Strengths and Difficulties Questionnaire (SDQ; k = 6), the Spence Children's Anxiety Scale (SCAS, k = 5), and the Emotion Regulation Checklist (ERC; k = 3). No studies considered the remission of diagnoses. We constructed one measure of *child externalizing behavior* (derived from, e.g., ECBI) and one of *child internalizing difficulties* (derived from, e.g., the SCAS), which are constructs commonly used in research of child and adolescent mental health studies (see Supplementary material for further details).

Twenty-nine studies measured change in parental mental health and behavior. A total of 30 measures were used in these studies. However, 19 measures (63%) were not used in more than one study, and eleven (37%) were used in at least two studies. The most common measures were the Parent/Maternal Emotional Style Questionnaire (PESQ/MESQ; k = 14), the Difficulties in Emotion Regulation Scale (DERS; k = 12), the Coping with Children's Negative Emotions Scale (CCNES; k = 12), the General Health Questionnaire (GHQ; k = 8), Parenting Sense of Competence (PSOC; k = 4), and the Emotions as a Child Scale (EAC; k = 4), in addition to the use of video recordings in data collection (k = 3). Several of the measures focus on different perspectives on emotion awareness or emotion coaching within parenting practices. We constructed a general measure of *parent mental health* and a general measure of *parent behavior* (capturing parents' involvement, reactivity, and warmth toward their children), constructs we believe all clinicians and researchers within psychotherapy would assess as important aspects to consider when investigating change in therapy. The construct of *parent mental health* (derived from, e.g., GHQ) included self-, partner-, and assessor-based ratings, while the construct of *parent behavior* (derived from, e.g., the Alabama Parenting Questionnaire) consisted of self-assessment questionnaires. In addition, we constructed *emotionally oriented parenting*, a construct focusing on the main element in the interventions we included in this study, as well as how parents relate to their own and their children's emotions. *Emotionally oriented parenting* (derived from, e.g., the PESQ, CCNES, and DERS) included self-assessment measures, as well as outsider observer ratings. Supplementary Table 1 provides a detailed description of what measures are included in each construct.

### 2.6. Statistical analysis

For all outcomes of interest, meta-analyses were conducted to estimate the effect of the interventions using a Robust Bayesian meta-analysis (RoBMA; Maier et al., 2022). This differs from our original intention to perform a simple random-effects model. A limitation of classic random-effects models, however, is that it is difficult to differentiate between study heterogeneity and publication bias. Second, publication bias cannot be reliably estimated if few studies are included (Maier et al., 2022). To avoid such limitations, the RoBMA was preferred. The RoBMA was chosen because it avoids several of the pitfalls associated with classic methods of assessing publication bias, such as low power in detecting publication bias, researcher intentions affecting the detection of publication bias, and study heterogeneity affecting measures of publication bias (Maier et al., 2022). The RoBMA estimates the weighted average of models assuming the presence vs. the absence of an effect, fixed vs. random effects, and the presence vs. the absence of publication bias (Maier et al., 2022). In other words, the RoBMA consists of three levels. The first level investigates whether an effect is present or not. The second level investigates whether the included studies measured the same underlying effect and are similar (fixed effects) or measured some degree of an underlying effect and differ in several ways (random effects). The final level of the RobMA investigates the hypothesis that publication bias is present or absent. This level tests publication bias from a purely statistical point of view (e.g., can we observe *p* > 0.05 in the sample) and does not account for more general biases (i.e., program developers conducting research, as assessed by the Robins-I tool mentioned above). As an assessment of the homogeneity of the effect sizes, we calculated the τ -statistic. Publication bias was assessed through a robust meta-analysis and described in terms of how probable the hypothesis of publication bias was, as compared to the hypothesis of a real effect. The Bayes factor (BF) is the main outcome and represents the amount of evidence in favor of either of the hypotheses.

All models were run using three chains; 2,500 burn-in iterations; and a minimum of 5,000 iterations. Sampling continued until an effective sample size of 5,000 and an *R*^2^ value below 1.02 were reached. Default priors were used (Bartoš et al., 2022), which represented a stance of equipoise across all aspects of the meta-analysis (e.g., the presence of an effect and the absence of an effect are both plausible hypotheses). The results are in favor of the presence vs. the absence of an effect, a fixed vs. a random effect, and the presence vs. the absence of publication bias. These are expressed as Bayes factors (BFs), with values between 1/3 and 3 indicating a lack of evidence, 3–10 moderate evidence, 10–30 strong evidence, 30–100 very strong – and >100 extremely strong evidence (Stefan et al., 2019). We present BFs with two decimals up to 1,000, and above this value, we report >1,000. All results are reported using 95% credibility intervals (95% CrI), which describe where the estimated parameter is located with 95% probability. When describing the heterogeneity of the meta-analysis, it is described as either large (2 *τ ≥ δ) or small (2 *τ ≤ δ), where δ is the estimated effect size. This categorization helps identify cases in which the heterogeneity is so large that it is reasonable to expect negative findings from new studies (Spineli and Pandis, 2020).

## 3. Results

### 3.1. Selection and inclusion of studies

Our search identified 8,272 studies. After abstract reading, full-text reading, and citation searching, 33 studies were included. Two of the identified studies were qualitative (Lambie et al., 2020; Ansar et al., 2021), whereas one study included qualitative descriptions (Wilhelmsen-Langeland et al., 2020), in addition to a primary focus on quantitative data. One study (Mastromanno et al., 2021b) consisted of three in-depth case descriptions taken from a connected Randomized Controlled Trial (RCT) study (Mastromanno et al., 2021a) and was therefore excluded from the meta-analysis. One large study focused on parents of adopted children (Rolock et al., 2021), which reported extreme effect sizes (Cohens *d* > 5). Because of the selective population, effect sizes were considerably higher than those normally found in psychotherapy, and because of the fact that we were not allowed access to the raw data, this primary study was excluded from the meta-analysis.

Seventy-six percent (k = 25) of the studies investigated different versions of the Tuning into Kids (TIK) treatment. Twelve percent (k = 4) of the studies investigated the closely connected Emotion-Focused Skills Training (EFST) and Emotion-Focused Family Therapy (EFFT), while each of the remaining 12% (k = 4) of studies investigated other types of emotionally oriented parental interventions. Of the studies that investigated different versions of TIK, the developer Havighurst was among the authors in 23 of the 25 studies. These were also among the primary studies to which we were not provided access to data.

### 3.2. Characteristics of included studies

Among the 33 studies, seventeen (52%) were conducted in Australia, and five were conducted in Norway (15%). Publication years ranged from 2004 to 2022, with 91% of articles being published post-2010 (k = 30).

Twenty-three (70%) of the studies had some type of randomized controlled design comparing experimental cases either to a waitlist (k = 14) or controls (k = 9). The follow-up duration varied. Some studies measured pre- to post- with only 2 days between the measurement points, while others involved long-term follow up. The maximum follow-up time was 12 months. A total of 1,903 participants' data have been collected. All participated in emotionally oriented parental interventions. Only one of the studies was pre-registered; however, it did not fully follow the protocol. The children included in the studies were from 1.5 to 18 years old. However, 24 (73%) studies had narrow age spans of 2–3 years, while two studies had age spans of more than 10 years. Most studies included children between 3 and 6 years (k = 17, 52%), one study included only toddlers (1.5–3 years old), and eleven studies included children above 10 years of age. All, except for one, of the primary studies had sample sizes smaller than 125 participants. More specifically, ten primary studies had sample sizes between 50 and 100, and eight primary studies had sample sizes ranging from 100 to 150 individuals. Only one study had more than 125 participants (Rolock et al., 2021). However, that study was excluded from the meta-analysis for reasons explained above.

Nine of the studies were assessed as having severe bias issues, while the remaining studies were assessed as having moderate bias. Because there was no evidence of research with a critical risk of bias, no studies were excluded from the analysis based on the risk of bias evaluation. The risk of bias was assessed as *severe* when there were (a) the selective choice of measures (only selected subscales from a measure), (b) measures with poor psychometrics, (c) allegiance (e.g., at least one of the authors was the developer or had financial interest in a positive outcome of the study), and (d) poor procedures for handling missing data.

#### 3.2.1. Reporting strategies

Most of the measures relied on parent self-report, but nine studies (27%) included teachers' or other outside observers' reports. Only four studies, of which three were based on the same sample, included children's self-reports.

### 3.3. Meta-analysis

**Figure 2:**
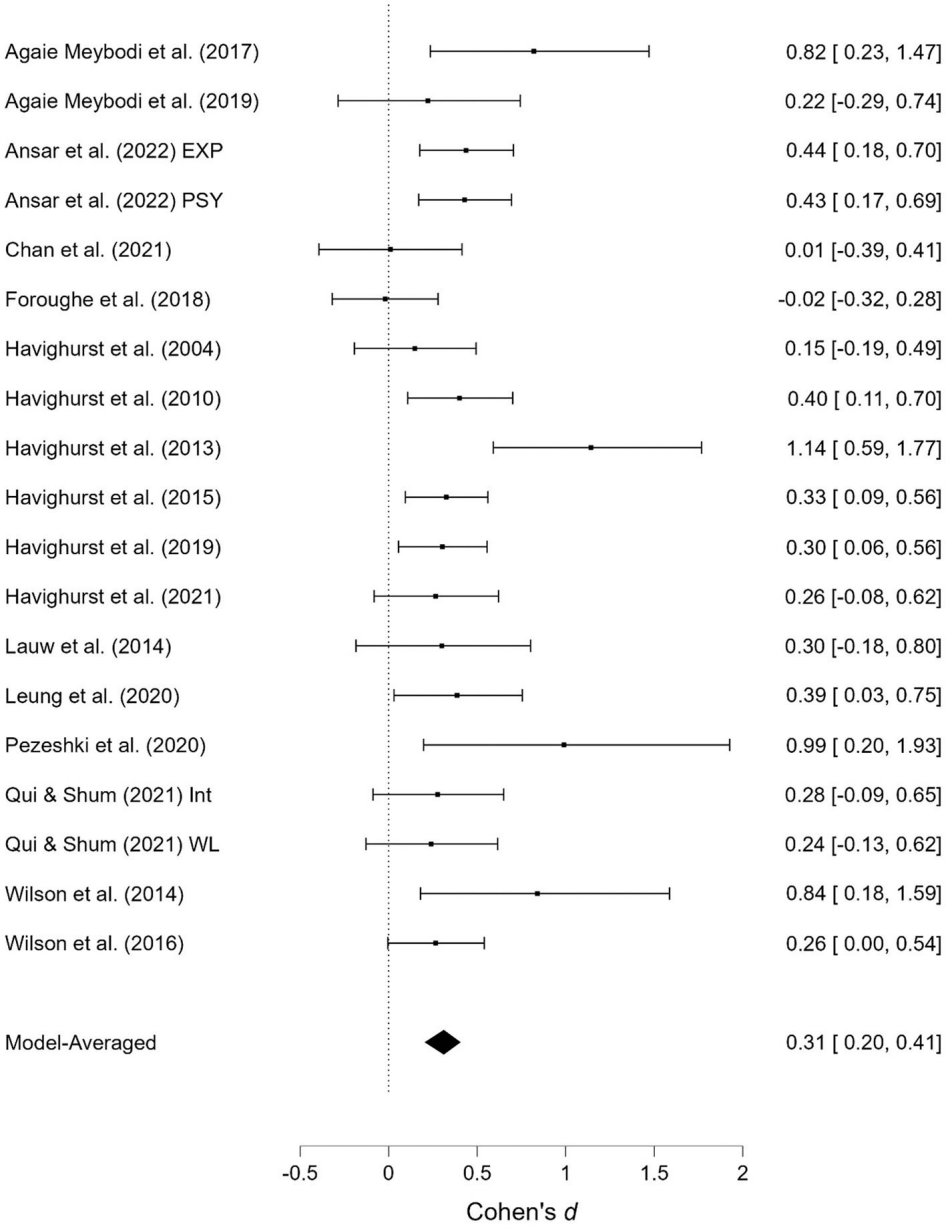
Child externalizing behavior at post-treatment.

**Figure 3:**
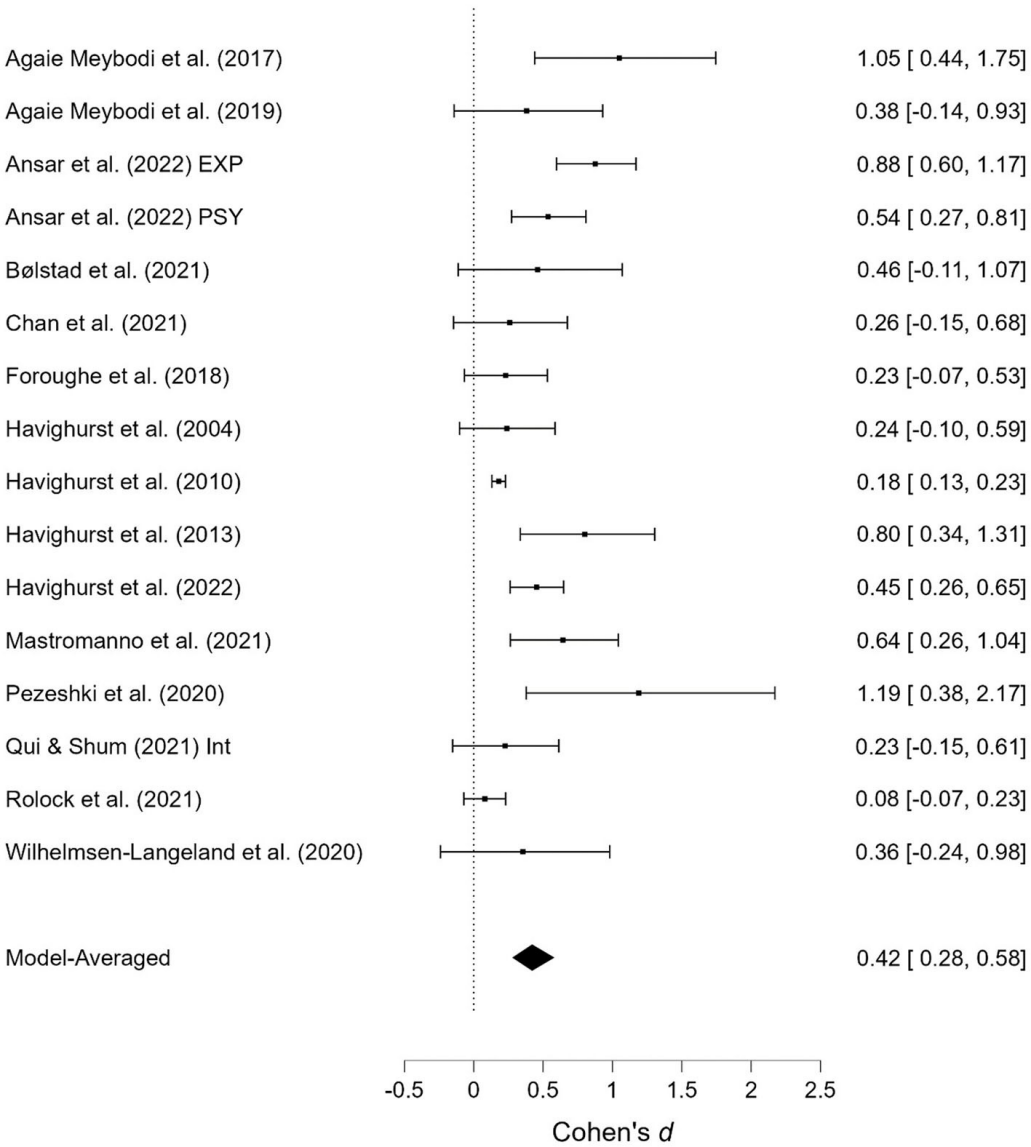
Child externalizing behavior at follow-up.

**Figure 4:**
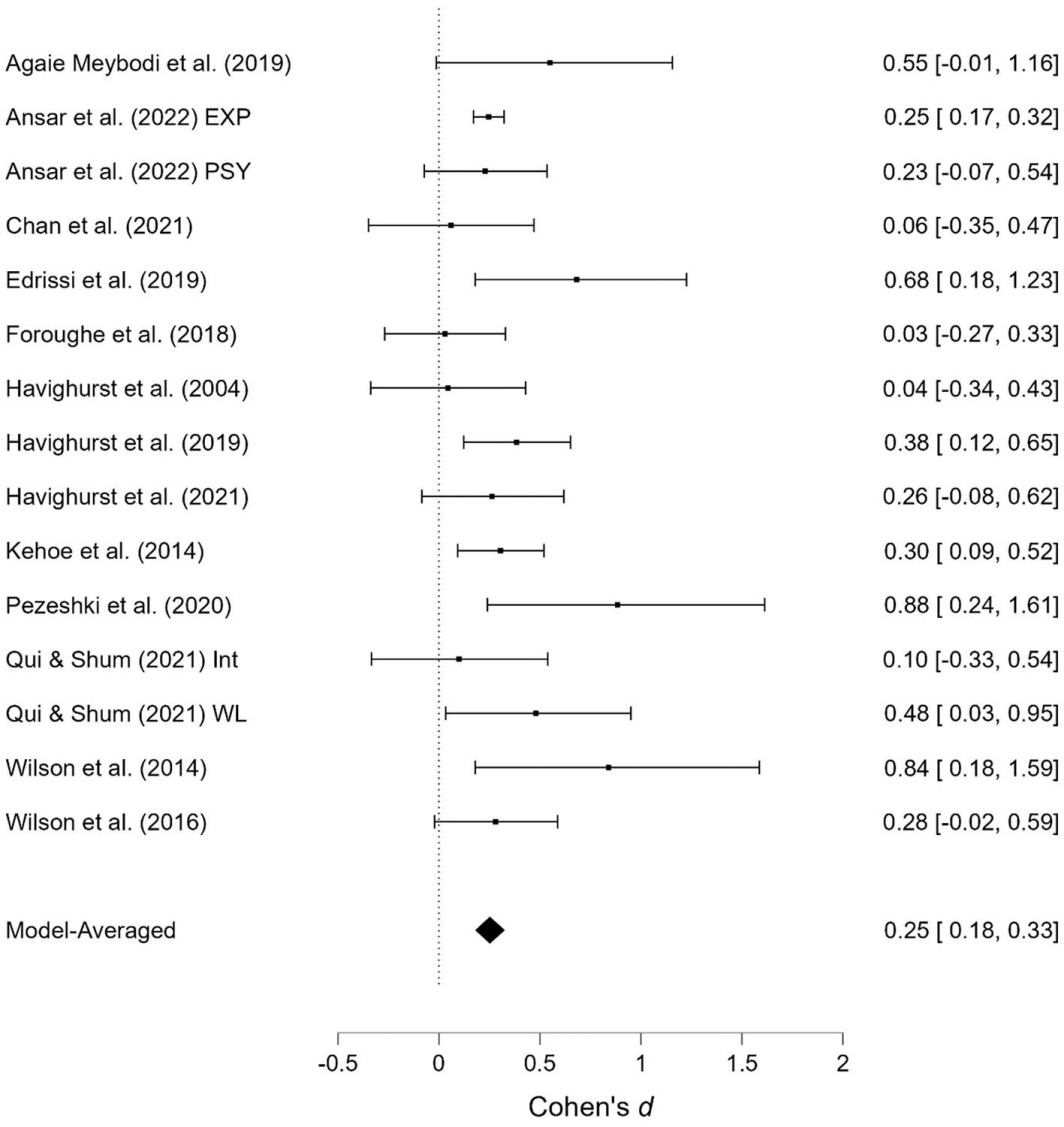
Child internalizing difficulties at post-treatment.

**Figure 5:**
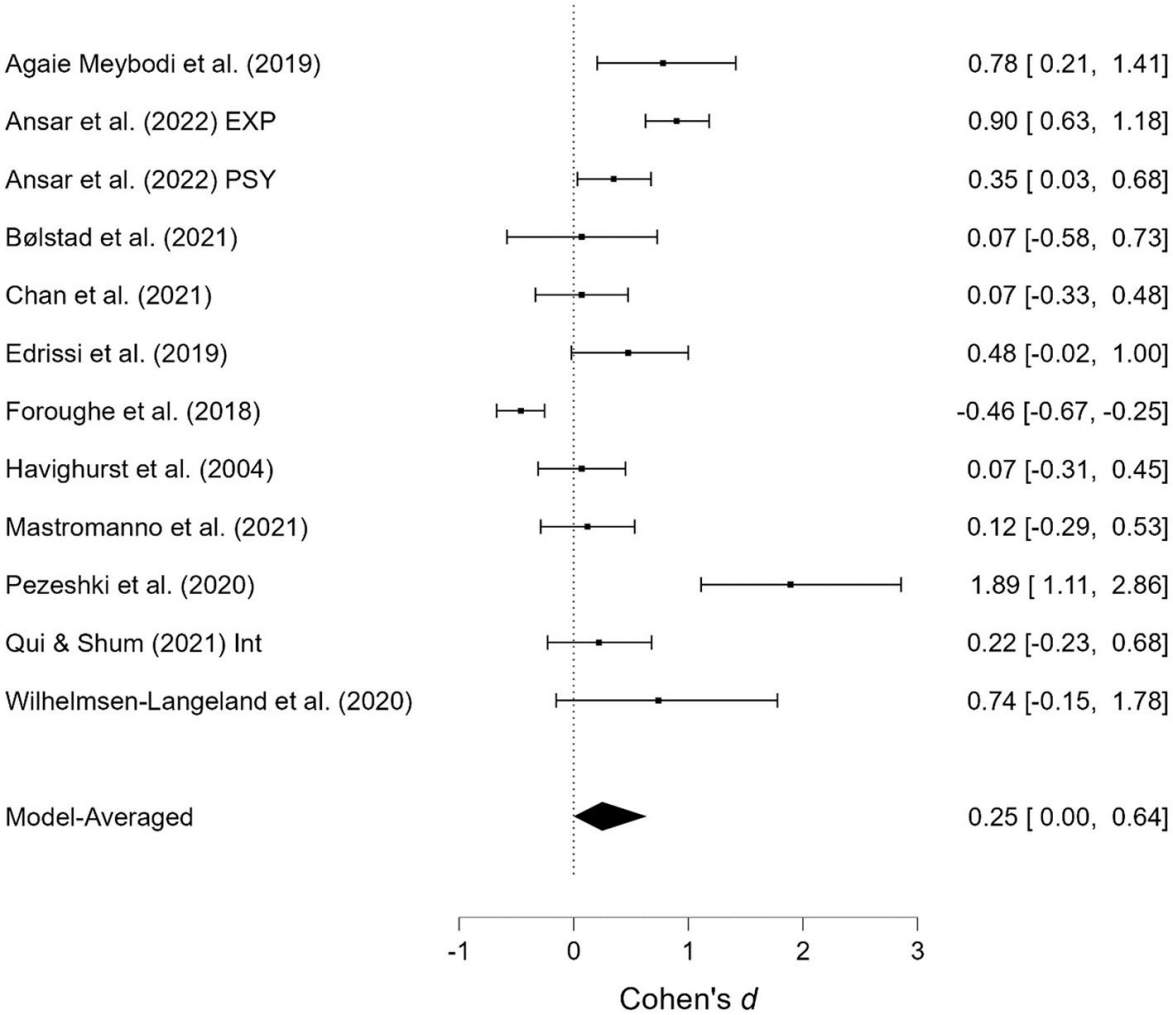
Child internalizing difficulties at follow-up.

**Figure 6:**
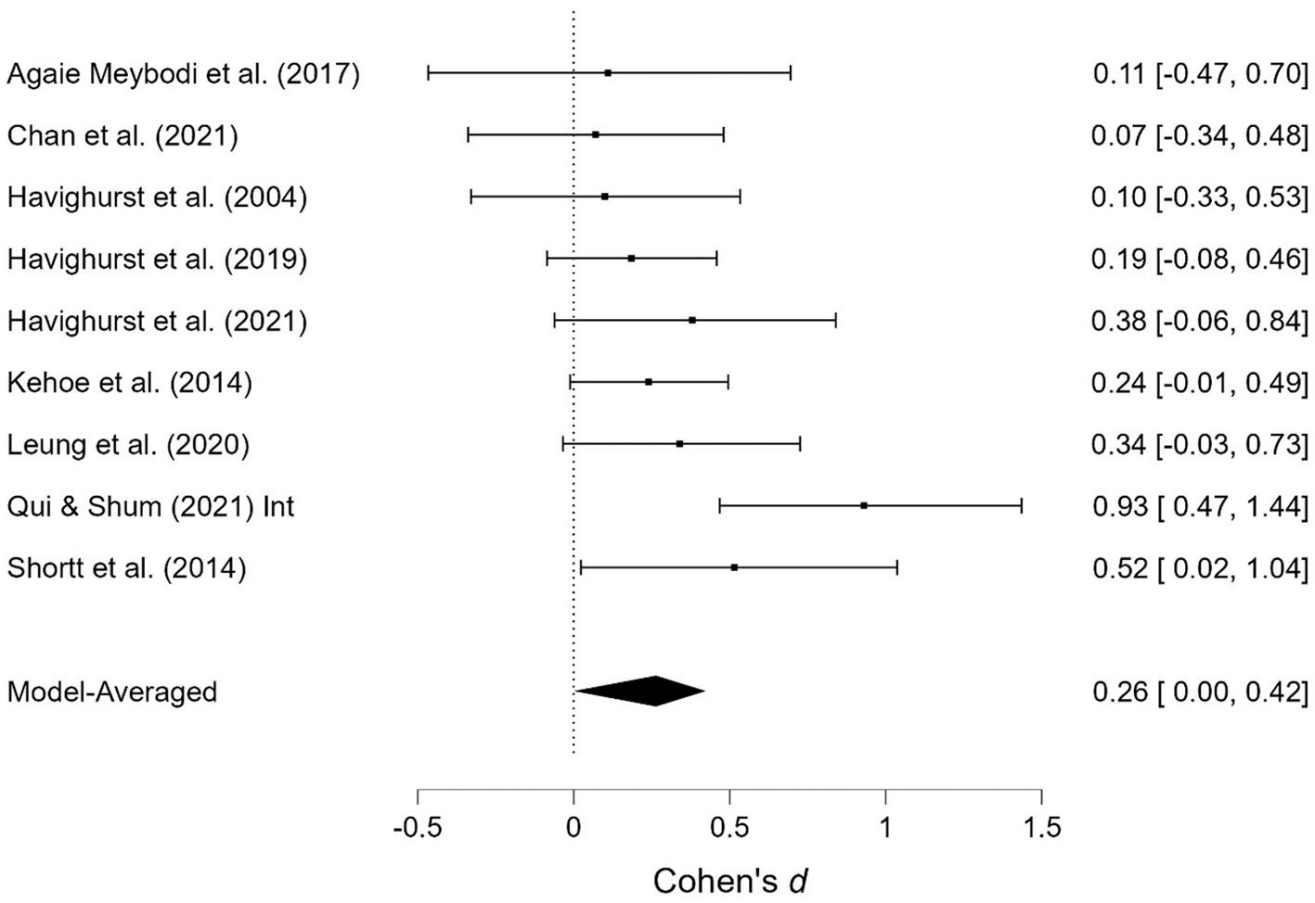
Parental mental health at post-treatment.

**Figure 7:**
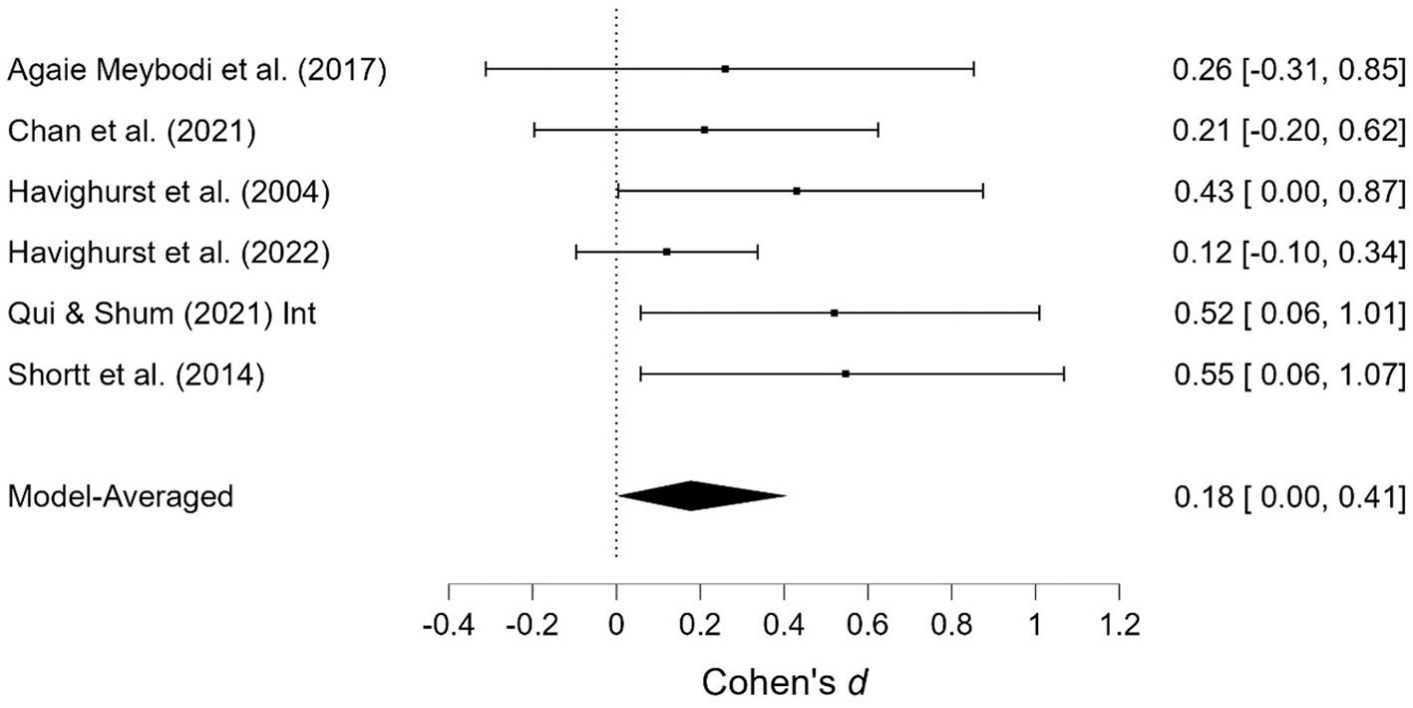
Parental mental health at follow-up.

**Figure 8:**
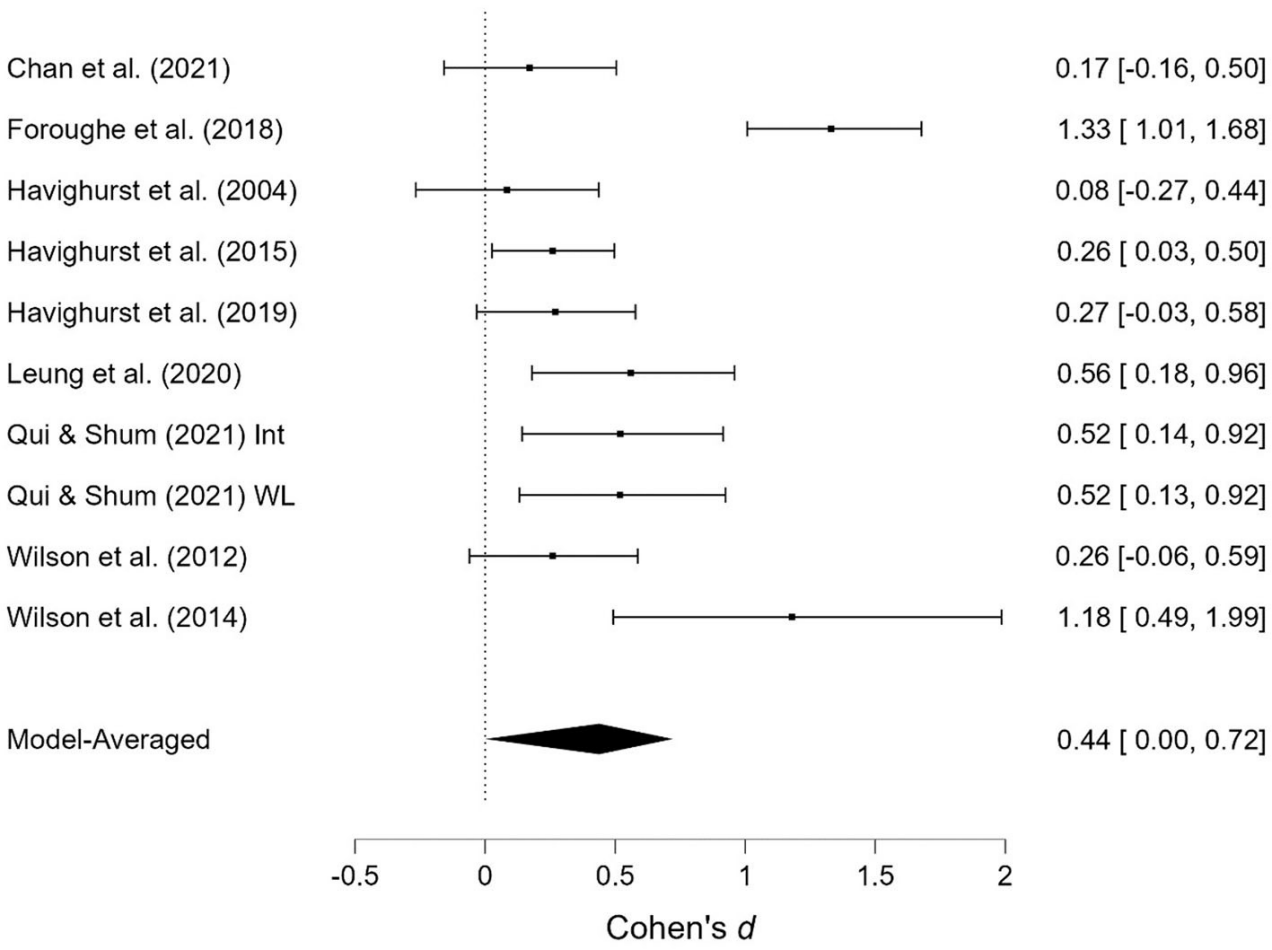
Parenting behavior at post-treatment.

**Figure 9:**
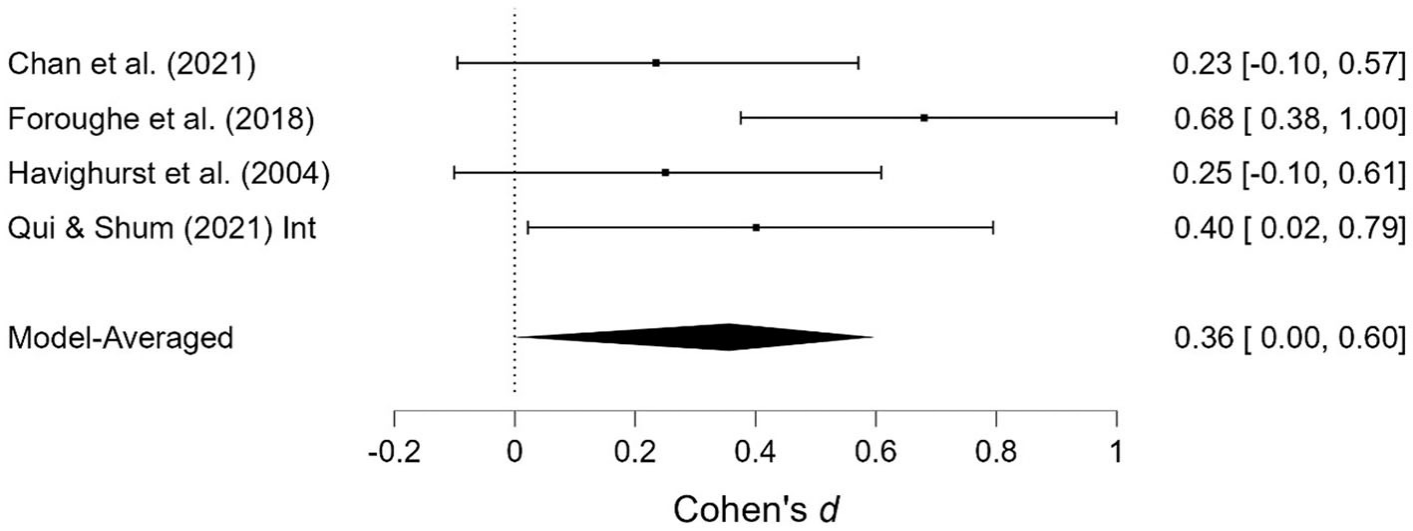
Parenting behavior at follow-up.

**Figure 10:**
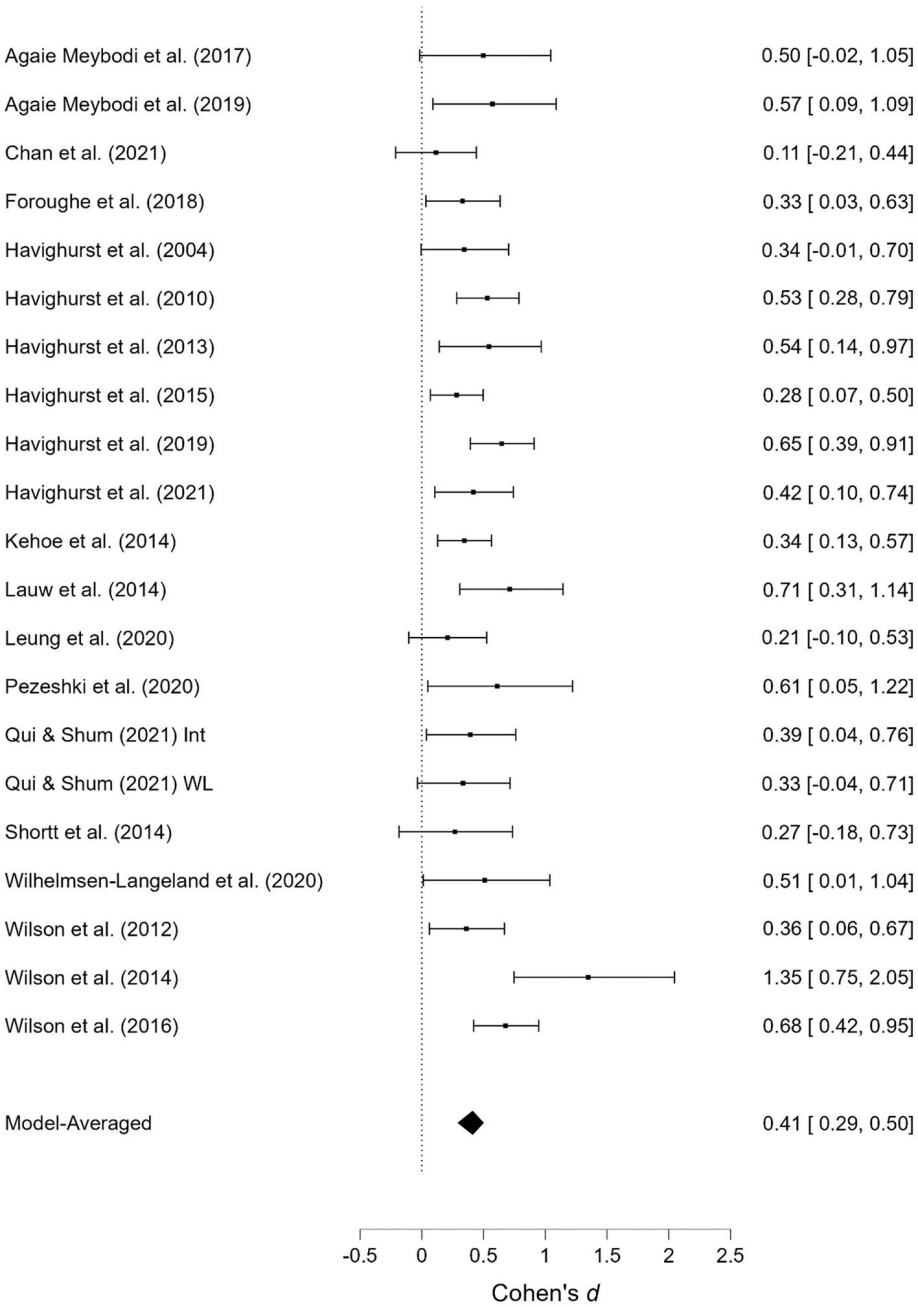
Emotionally oriented parenting at post-treatment.

**Figure 11:**
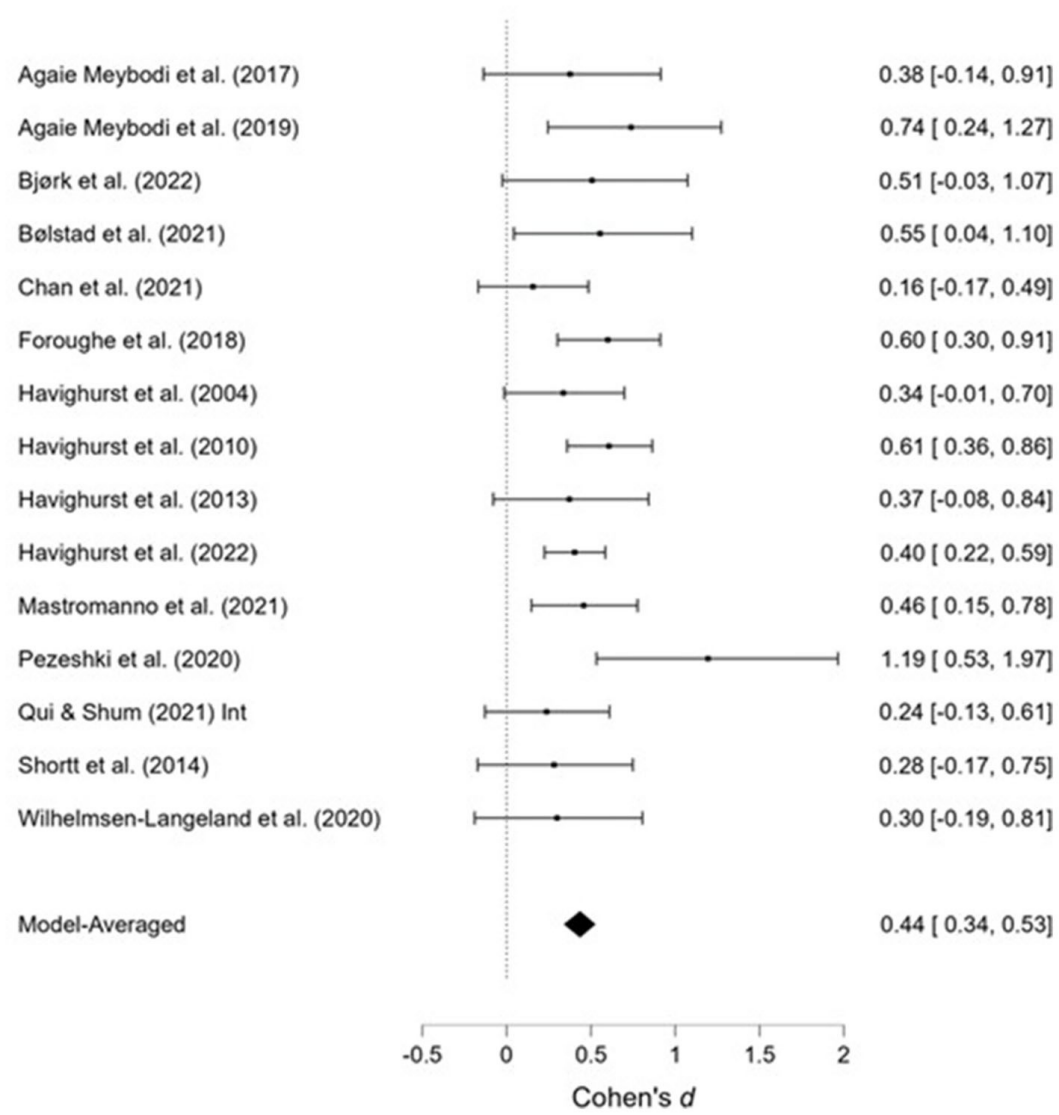
Emotionally oriented parenting at follow-up.

As shown in Table 1, several studies were based on the same sample but provide different outcomes. When presenting the results of the meta-analyses, each of these studies is presented individually; however, when calculating the overall effect size, they are counted as a single study. Two studies contained two samples receiving emotionally oriented parental interventions, and the results for each of these are presented separately. The results of the meta-analyses are presented in Figures 2–11. *Children's externalizing behaviors* are presented in Figures 2, 3, *children's internalizing difficulties* are presented in Figures 4, 5, *parental mental health* is presented in Figures 6, 7, *parenting behavior* is presented in Figures 8, 9, and *emotion-focused parenting* is presented in Figures 10, 11.

The use of Bayesian analyses allowed us to test whether the data provided more evidence for one hypothesis as compared to another. For each construct, we performed three tests. As mentioned above, we first tested whether there was evidence for the presence of an effect vs. evidence for the absence of an effect. Secondly, we tested whether there was evidence of fixed vs. random effects. Thirdly, we tested whether there was evidence of the presence vs. the absence of publication bias. In the cases in which there was lack of evidence in any direction, we have presented the results in the Supplementary material and omitted the results in the text. In those cases in which there was substantial evidence in favor of one hypothesis, the BF indicates the degree of evidence for this hypothesis vs. the opposing one.

#### 3.3.1. Child externalizing behavior

The effect of the interventions on *child externalizing behavior* post-treatment and at follow-up was estimated at d = 0.31, 95% CrI (0.20, 0.41); 0.42, 95% CrI (0.28, 0.58), with a small amount of heterogeneity between studies post-treatment [τ = 0.03, 95% CrI (0.00, 0.18)] and large heterogeneity at follow-up [τ = 0.22, 95% CrI (0.12, 0.37)]. There was extremely strong evidence of an effect (BF > 1,000; BF > 1,000). There was extremely strong evidence in favor of random effects model vs. a fixed effects model (BF > 1,000) at follow-up, and some degree of evidence for the absence of publication bias (BF = 0.23) at follow-up.

#### 3.3.2. Child internalizing difficulties

The effects of the interventions on child internalizing difficulties post-treatment and at follow-up were estimated at d = 0.25, 95% CrI (0.18, 0.33); d = 0.25, 95% CrI (0.00, 0.64), with a small amount of heterogeneity between studies [τ = 0.01, 95% CrI (0.00, 0.13)] post-treatment and a large amount at follow-up [τ = 0.51, 95% CrI (0.29, 0.85)]. There was strong evidence of an effect post-treatment (BF > 1,000). There was evidence in favor of a fixed effects model (BF = 0.20) post-treatment, but strong evidence in favor of random effects model at follow-up (BF > 1,000). There was weak evidence indicating the absence of publication bias (BF = 0.33) at follow-up.

#### 3.3.3. Parental mental health

The effects of the interventions on *parental mental health* post-treatment and at follow-up were estimated at d = 0.26, 95% CrI (0.00, 0.42); d = 0.18, 95% CrI (0.00, 0.41), with a small amount of heterogeneity between studies [τ = 0.05, 95% CrI (0.00, 0.30); τ = 0.05, 95% CrI (0.00, 0.28)]. There was strong evidence of an effect (BF = 30.50) post-treatment but only weak evidence for such at follow-up (BF = 3.32).

#### 3.3.4. Parenting behavior

The effects of the interventions on *parenting behavior* post-treatment and at follow-up were estimated to d = 0.44, 95% CrI (0.00, 0.72); d = 0.36, 95% CrI (0.00, 0.60), with a large amount of heterogeneity between studies [τ = 0.35, 95% CrI (0.18, 0.64)] post-treatment and a small amount at follow-up [τ = 0.10, 95% CrI (0.00, 0.50)]. There was strong evidence of an effect post-treatment (BF = 25.54) and at follow-up (BF = 10.81). There was extremely strong evidence in favor of a random effects model (BF > 1,000) post-treatment.

#### 3.3.5. Emotionally oriented parenting

The effects of the interventions on *emotionally oriented parenting* post-treatment and at follow-up were estimated at d = 0.41, 95% CrI (0.29, 0.50); d = 0.44, 95% CrI (0.34, 0.53), with a small amount of heterogeneity between studies [τ = 0.06, 95% CrI (0.00, 0.20); τ = 0.02, 95% CrI (0.00, 0.16)]. There was extremely strong evidence of an effect (BF > 1,000; BF > 1,000).

#### 3.3.6. Overall meta-analysis

Overall, the meta-analysis showed that, post-treatment, there was evidence of an effect of the interventions on all constructs. For *parent behavior*, post-treatment, there was evidence for a large amount of heterogeneity between studies, suggesting that (a) the effect on parent behavior differed between the interventions, (b) measuring parent behavior is difficult when using self-assessment, or (c) the construct was capturing not one but a diverse set of perspectives. Post-treatment, all constructs lacked evidence for or against publication bias, indicating that there is insufficient evidence to conclude that there was or was not publication bias post-treatment.

At follow-up, there was evidence of an effect of the interventions on parent mental health, parent behavior, emotion-focused parenting, and child externalizing behavior. There was no evidence for an effect on child internalizing difficulties, indicating that there is insufficient evidence to conclude that an effect exists or that, in fact, an effect does not exist. In addition, there was a large amount of heterogeneity between studies regarding the construction of child externalizing behavior and child internalizing difficulties. At follow-up, there was also evidence in favor of the absence of publication bias on these two constructs. For the other measures, there was a lack of evidence for or against publication bias. In sum, all measures used to assess the overall level of publication bias indicated an absence of publication bias in the field of emotionally oriented parental programs research (BF = 0.11).

## 4. Discussion

### 4.1. Effect sizes and immediate and delayed effect

In this meta-analysis based on 29 outcome studies, we observed a post-treatment effect for all included constructs: *child externalizing behavior, child internalizing difficulties, parental mental health, parenting behavior, and emotionally oriented parenting*. The credibility intervals for the effect of child measures did not include zero and were relatively narrow, with mean effect sizes of 0.25 and 0.31, which are considered small effect sizes (Cohen, 1988).

The results indicate that the effects on the children increase at follow-up. This is particularly true for child externalizing behavior, but because the longest follow-up period was 12 months, we are unable to determine whether this development continues for longer than this. Plausible explanations exist regarding why the effect is higher on children's externalizing behavior as compared to internalizing difficulties. While externalizing behavior in children is highly visible thus making it easier for parents and teachers to observe it, it may be more difficult to report internalizing difficulties (Margherio et al., 2019). Therefore, collecting data directly from children could identify internalizing difficulties and possible change within this domain (Margherio et al., 2019). However, few of the included studies incorporated child reports. It may also be that children with internalizing difficulties benefit more significantly from direct therapy than parental programs (Monga et al., 2015; Creswell et al., 2017). However, if the increase at follow-up is due to the parents' new ways of interacting with their children, which is the primary goal of parental interventions, the identified effect sizes indicate that the parents have adapted, to some degree, during the interventions and continue to act in accordance with their newly acquired competence after the program has concluded. This notion is supported by the results of this meta-analysis regarding the effect on emotionally oriented parenting, which is the primary emphasis of the interventions. The analyses revealed that the effect was somewhat greater at follow-up than post-treatment, a finding that is in line with studies of parent based CBT treatment for child anxiety (Brown et al., 2017). The fact that the effect increased at follow-up may suggest that parents translating the principles and abilities acquired during the interventions into their everyday parenting may be a gradual process, similar to how parents who move from one culture to another gradually change their parenting style (Glick et al., 2012; Bornstein et al., 2020). Thus, the delayed effect of the interventions on children's externalizing behavior may be partially attributable to the fact that it takes time for parents to adapt to a new behavior and for children to accept and positively respond to this new or reinforced emotionally oriented parenting behavior.

The effects on the children were indirect, as they did not directly participate in the interventions. An indirect approach to effect changes in the child is often used for small children and may also be preferable for older children and youths in instances in which it would be considered unfeasible or even stigmatizing for them to receive direct mental health interventions. Moreover, in accordance with family systems theories (Kerr and Bowen, 1988; Johnsen and Torsteinsson, 2012; Haefner, 2014), it is plausible to assume that the changes adopted by parents will also benefit their other children. If the effects of parental interventions reach several family members, they may help change the entire family system.

In relation to this discussion of parental change and how it may interact with change in children, it is important to comment on the three parental constructs used in this study. The *emotionally oriented parenting* construct combines more than ten measures, but the meta-analysis does indicate a small amount of heterogeneity. This suggests that these measures all tap into the same area of parenting and that the effects of the interventions are similar in the included studies. *Parental behavior* shows a large amount of heterogeneity post-treatment, and we recognize that, when creating this construct, we combined measures aimed at tapping into various aspects of parental behavior. However, at follow-up, the result identified only small amounts of heterogeneity, suggesting that the studies identified similar effects on different aspects of parent behavior or that the measures used in these studies capture the same concept with similar effects. This leads to a related issue highlighted by this study: the fact that the field employ such a variety of measures. Indeed, most measures were only used in one study. We therefore recommend that the field select a few psychometrically robust metrics for use in future research. This would both strengthen such studies but also make it easier to conduct meaningful meta-analyses. The measures most often used for parental change were the MESQ/PESQ and DERS. However, the MESQ has only one psychometric study as its basis (Lagacé-Séguin and Coplan, 2005), and it appears that the PESQ, an extended version of the MESQ, has none. The DERS on the other hand, has several psychometrical studies as its basis (e.g., Bjureberg et al., 2016; Hallion et al., 2018), confirming the validity and reliability of the measure. The measures most often used in assessing change in children's difficulties were the ECBI, CBCL, and SDQ, All three have been shown to have good validity and reliability (Nakamura et al., 2009; Stone et al., 2010; Sorsa et al., 2019).

For measures of *parental mental health* and *parent behavior*, our study identified effect sizes ranging from 0.20 to 0.44, which are considered small. A reasonable hypothesis is that the parents engaged in the programs not due to difficulties with their own mental health but, rather, due to struggles with their child or the interplay with the child. More research on such programs is needed to draw further conclusions regarding actual changes in parental mental health. One might argue that even a small effect on the mental health of parents can be of great importance for the child and the parent-child interaction. The results at follow-up indicate that parents benefit most while they are in the program, and we observed that the effects on *parenting behavior* and *parental mental health* diminish at follow-up. While participating in a parenting program, there is great focus and encouragement on the part of the facilitator and the other group members on being particularly sensitive to emotional cues within themselves and the child, in addition to practicing new behavioral skills. This may create immediate experiences of hope, which again may foster immediate relief and behavioral changes. The situation then returns to normal after some time.

Although this meta-analysis identified an effect of emotionally oriented parental interventions, one may question the clinical significance of these findings considering that many of the studies recruited their participants from community settings. Even if several studies included only children with specified levels of problems measured with, for example, the CBCL, only one of the included studies collected its sample from a clinical setting, such as a hospital or mental health clinic (Havighurst et al., 2013). This represents a gap in the literature and should encourage additional studies in clinical settings so as to elucidate the effects of these interventions on clinical populations.

From a statistical perspective, the fact that one study indicated a negative effect at follow-up is considered a strength, as negative outcomes are typically not recorded despite the fact that they are predicted when numerous studies address the same issue (Chambers, 2017). The willingness to publish all results is vital for legitimacy and transparency in a growing field.

### 4.2. Methodological issues

In many of the studies, the developer of the program was involved as a trainer and researcher. This can be argued to be a strength because it helps ensure that the program was delivered as described in the literature. However, it is likely that a developer has a strong preference for their program to be deemed beneficial, and several studies have identified that including the developer as part of the research team increases the reported effect, with medium to large effects, which is also commonly referred to as researcher allegiance (Munder et al., 2013; Wampold and Imel, 2015). A large study of RCTs within psychotherapy identified that the allegiance effect was even stronger when the developer of the preferred treatment was one of the authors and supervised or trained the therapists (Dragioti et al., 2015). However, this systematic review focuses on newly developed programs, and it may be typical at that stage for the developer to be involved in many of the research projects. As the programs become more widely used, this will likely change.

In this review, a number of studies were evaluated as having a high risk of bias due to the fact that they presented data from only a selection of the applied scales. We do not know why the results for the other scales were not presented. However, it is plausible that there were no significant outcomes or, perhaps, even negative outcomes for the other measures. The study of Simmons et al. (2016) exemplifies this problem by demonstrating that it is more likely for a researcher to find false evidence that an effect exists than to find correct evidence that it does not exist. The pre-registration of studies and the reporting of all results will benefit the reliability of this field of research.

In many of the identified studies, the rate of missing data was low (< 10%), and the *last one carried forward* method was used to handle the data. If participants did not have the same favorable experience as others who participated in the same intervention, they may be reluctant to submit answers, and the *last one carried forward* method removes those negative outcomes, which would have decreased the effect of the treatment. However, that method favors no change, and if non-responding participants were similar to those providing their responses, they would have increased the calculated effect of the treatment. One study in particular handles missing data in a more sophisticated manner by modeling the outcome depending on multiple missingness assumptions (Ansar et al., 2022). When missingness exceeds 5%, full information maximum likelihood (FIML) is suggested; however, multiple imputation is generally preferred (Jakobsen et al., 2017).

### 4.3. Recommendations for future studies

We recommend that future studies should be pre-registered, use measures with high levels of validity and reliability, and share their data publicly. We requested data from all studies, but we only received five datasets. Within the field of psychology and psychotherapy research, the failure to openly share data is a common issue (Chambers, 2017). Less than 30% of researchers in Wicherts et al.'s (2006) analysis of major American Psychological Association (APA) journals shared their data. Wicherts et al. (2011) found that papers in which the authors did not provide their data were more than twice as likely to misreport *p*-values. Public data deposition should be the standard for future studies if this discipline is to advance and meet reliability demands. Fortunately, this is becoming the standard for publication in a larger portion of journals.

There is a need for more studies using rigorous designs, such as RCT designs, to compare emotionally oriented parental interventions to other interventions. Even if our study did not find strong evidence for publication bias in the included studies, we encourage researchers not closely related to developers to conduct effectiveness studies. Because family systems theory predicts that changes in parental behavior and understanding will impact the entire family system, we recommend that future studies include siblings in the assessments. Furthermore, we have identified a need for studies that investigate the effects on children, preferably children with defined diagnoses, within a clinical context. There is also a need for studies that investigate the mechanisms of change involved in parental interventions and thus empirically testing various aspects of the theories of change put forth in the literature.

## Data availability statement

The original contributions presented in the study are included in the article/Supplementary material, further inquiries can be directed to the corresponding author.

## Author contributions

RZO, LS, JS, CRF, IS, and TB contributed to conception and design of the study. SR and RZO conducted the protocol and the searches. RZO, LS, CRF, and IS performed the screening and risk of bias analyses. RZO and TB performed the data extraction. TB conducted the meta-analyses. RZO wrote the first draft of the manuscript with help from JS for the introduction. All authors contributed to manuscript revision, read, and approved the submitted version.
