# Supplementary material for: Effects of emotionally oriented parental interventions: a systematic review and meta-analysis
Source: Front Psychol. 2023 Jul 13;14:1159892. doi: 10.3389/fpsyg.2023.1159892 (PMC10374204; doi:10.3389/fpsyg.2023.1159892)
Supplement: Supplementary file 1 [file Table_1.DOCX]

Supplementary Material

**Effects of Emotionally Oriented Parental Interventions: A Systematic Review and Meta-analysis**

**Rune Zahl-Olsen, Linda Severinsen, Jan Reidar Stiegler, Carina Ribe Fernee, Indra Simhan, Sondre Sverd Rekdal, Thomas Bjerregaard Bertelsen**

*** Correspondence:** Rune Zahl-Olsen: rune.zahl-olsen@sshf.no

# Supplemental Table 1 - Measures used in each Construct

| **Child externalizing behavior** | **Child Internalizing difficulties** | **Parental mental health** | **Parenting behaviour** | **Emotionally oriented parenting** |
| --- | --- | --- | --- | --- |
| ECBI -Child behavior | ERC - Child Liability | GHQ | PSI-SF parent child dysfunction | PESQ - Emotion dismissing |
| ECBI - Child emotion regulation | CBCL- Internalizing | PSI-SF-parental distress | PvA - parental self-efficacy | DERS - total |
|  |  |  |  |  |
| CBCL - Externalizing scale | PAS-R Child total anxiety | DASS - total | CRQ - Warmth | ERQ - Expressive suppression |
|  |  |  |  |  |
| ERC - Child liability/negativity | ERC - Child Adaptive Emotion regulation | CESD | FCS - family conflict | CTS - Parent emotion blocks |
| SDQ -total difficulties | SDQ - emotional problems | Measured cortisol | Parenting scale - over-reactivity | CCNES - problem focused reactions |
|  |  |  |  |  |
| SDQ - prosocial behavior | SCAS-P total anxiety |  | FCS - total | PESQ - Parent Empathy/Connection |
| SDQ - externalizing problems | CBCL- stress problems |  | APQ - positive involvement | EAC - Parent dismissing sadness |
| ERC - Child emotional regulation |  |  | Parental reactivity/anger | PSOC - total |
| BITSEA - Toddler behavior problems |  |  |  | PSOC - satisfaction |
| SDQ - Peer problems |  |  |  | PSOC - parent efficacy |
| SESBI - total |  |  |  | PRQ - relational frustration |
| TTS - child temperament |  |  |  | CCNES - punitive reactions |
|  |  |  |  |  |
| SESBI - intensity |  |  |  | PRFQ - IC |
|  |  |  |  |  |
| BPI - child behavior problems |  |  |  |  |
| CBCL - oppositional defiant problems |  |  |  |  |

Note: APQ= Alabama Parenting Questionnaire, BITSEA= The Brief Infant Toddler Social Emotional Assessment, BPI= Behavioral Problem Index, CBCL= Child Behavioral Checklist, CCNES= Coping with Children’s Negative Emotions Scale, CRQ= Child Rearing Questionnaire, CTS= The Caregiver Traps Scale, DASS= The Depression, Anxiety and Stress Scale, DERS= the difficulties in emotion regulation scale, EAC= Emotions as a child scale, ECBI= Eyberg Child Behavior Inventory, ERC = The Emotion Regulation Checklist, ERQ= Emotion Regulation Questionnaire, FCS= Family conflict scale, GHQ= General Health Questionnaire, PAS-R= The Preschool Anxiety Scale Revised, PESQ= Parent Emotional Style Questionnaire, PRFQ= Parental Reflective Functioning Questionnaire, PRQ= The Parenting Relationship Questionnaire, PSI-SF= Parenting Stress Index–Short Form, PSOC= Parenting Sense of Competence, PvA= Parent versus Anorexia Scale, SCAS-P= Spence Children’s Anxiety Scale-parent version, SESBI= the Sutter-Eyberg Student Behaviour Inventory, SDQ= Strengths and Difficulties Questionnaire, TTS= Toddler Temperament Scale.

# Supplemental Table 2 - lack of evidence for one hypothesis vs another

| Construct | Time | Effect vs no effect | fixed effect model vs random effect model | presence vs. absence of publication bias |
| --- | --- | --- | --- | --- |
|  |  |  |  |  |
| Parental mental health | post-treatment |  | BF=0.60 | BF=0.55 |
|  | Follow-up |  | BF=0.50 | BF=2.11 |
|  |  |  |  |  |
| *parenting behaviour* | post-treatment |  |  | BF= 0.45 |
|  | Follow-up |  | BF= 1.02 | BF= 0.49 |
|  |  |  |  |  |
| *Emotion focused parenting* | post-treatment |  | BF=0.94 | BF=1.18 |
|  | Follow-up |  | BF=0.37 | BF=0.51 |
|  |  |  |  |  |
| *child externalizing behaviour* | post-treatment |  | BF= 0.40 | BF= 0.82 |
|  | Follow-up |  |  |  |
|  |  |  |  |  |
| child internalizing symptoms | post-treatment |  |  | BF= 1.29 |
|  | Follow-up | BF = 2.33 |  |  |

# Supplemental Table 3 - Excluded studies

| **Reason** | | **Authors** | | **Year** | | **Title** | |
| --- | --- | --- | --- | --- | --- | --- | --- |
| 3 | | Altafim et al. | | 2021 | | Unpacking the Impacts of a Universal Parenting Program on Child Behavior | |
| 3 | | Arabi et al. | | 2020 | | The effect of emotion regulation training on family relationships of hyperactive children | |
| 1 | | Bloch et al. | | 2011 | | The Attachment Frame is the Thing: Emotion-Focused Family Therapy in Adolescence. | |
| 1 | | Cortell | | 2009 | | A pilot study of an emotion coaching and mindfulness program for parents of early adolescents | |
| 3 | | David et al. | | 2014 | | Efficacy of the Rational Positive Parenting Program for child externalizing behavior: Can an emotion-regulation enhanced cognitive-behavioral parent program be more effective than a standard one? | |
| 3 | Duncombe et al. | | 2016 | | Comparing an Emotion- and a Behavior-Focused Parenting Program as Part of a Multsystemic Intervention for Child Conduct Problems | |  |
| 2 | Franz et al. | | 2010 | | Effectiveness of an attachment-oriented parental training program for single mothers and their children: PALME. | |  |
| 3 | Gewirtz et al. | | 2019 | | Effects of the After Deployment: Adaptive Parenting Tools (ADAPT) intervention on fathers and their children: A moderated mediation model | |  |
| 3 | Havighurst et al. | | 2015 | | An emotion-focused early intervention for children with emerging conduct problems | |  |
| 1 | Havighurst et al. | | 2020 | | Emotion-focused parenting interventions for prevention and treatment of child and adolescent mental health problems: a review of recent literature. | |  |
| 3 | Herbert et al. | | 2013 | | A randomized controlled trial of a parent training and emotion socialization program for families of hyperactive preschool-aged children | |  |
| 1 | Herbert | | 2014 | | Parent training for families of hyperactive preschool-aged children | |  |
| 3 | Katz et al. | | 2020 | | An emotion coaching parenting intervention for families exposed to intimate partner violence | |  |
| 1 | Katz | | 2011 | | Tuning in to Kids' parenting programme improves parental emotional awareness, and child behaviour and emotional knowledge | |  |
| 4 | Lafrance Robinson et al. | | 2016 | | Emotion-Focused Family Therapy for Eating Disorders Across the Lifespan: A Pilot Study of a 2-Day Transdiagnostic Intervention for Parents. | |  |
| 3 | Loevaas et al. | | 2019 | | Does the transdiagnostic EMOTION intervention improve emotion regulation skills in children? | |  |
| 2 | Otterpohl et al. | | 2020 | | A German adaptation of tuning in to kids: Fostering emotion socialization strategies in German parents of preschool children | |  |
| 4 | Sabey et al. | | 2021 | | Processes and outcomes of an emotion-focused family therapy two-chair intervention for transforming problematic parenting patterns | |  |
| 3 | Shaffer et al. | | 2019 | | Let's Connect: A developmentally-driven emotion-focused parenting intervention | |  |
| 1 | Stavrianopoulos et al. | | 2014 | | Emotionally focused family therapy: Facilitating change within a family system. | |  |
| 4 | Strahan et al. | | 2017 | | Increasing parental self-efficacy with emotion-focused family therapy for eating disorders: A process model. | |  |
| 1 | Quinn | | 2020 | | Caregiver openness in emotionally focused family therapy: A critical shift. | |  |

Note: 1 = Dissertation/review/description only, 2 = Language (German), 3 = combined with other type of intervention, 4 = Child age

# Supplemental bibliography – included studies

Ansar, N., Hjeltnes, A., Stige, S. H., Binder, P. E., & Stiegler, J. R. (2021). Parenthood—Lost and Found: Exploring Parents’ Experiences of Receiving a Program in Emotion Focused Skills Training. Frontiers in Psychology, 1604. <https://doi.org/10.3389/fpsyg.2021.559188>

Ansar, N., Nissen Lie, H. A., Zahl-Olsen, R., Bertelsen, T. B., Elliott, R., & Stiegler, J. R. (2022). Efficacy of Emotion-Focused Parenting Programs for Children’s Internalizing and Externalizing Symptoms: A Randomized Clinical Study. *Journal of Clinical Child & Adolescent Psychology*, 1-17. <https://doi.org/10.1080/15374416.2022.2079130>

Aghaie Meybodi, F., Mohammadkhani, P., Pourshahbaz, A., Dolatshahi, B., & Havighurst, S. (2017). Reducing children behavior problems: a pilot study of Tuning in to Kids in Iran. *Iranian Rehabilitation Journal*, *15*(3), 269-276. <https://doi.org/10.29252/nrip.irj.15.3.269>

Aghaie Meybodi, F., Mohammadkhani, P., Pourshahbaz, A., Dolatshahi, B., & Havighurst, S. S. (2019). Improving parent emotion socialization practices: piloting tuning in to kids in iran for children with disruptive behavior problems. *Family Relations*, *68*(5), 596-607. <https://doi.org/10.1111/fare.12387>

Bjørk, R. F., Bølstad, E., Pons, F., & Havighurst, S. S. (2022). Testing TIK (Tuning in to Kids) with TEC (Test of Emotion Comprehension): Does enhanced emotion socialization improve child emotion understanding? *Journal of Applied Developmental Psychology*, *78*, 101368. <https://doi.org/10.1016/j.appdev.2021.101368>

Bølstad, E., Havighurst, S. S., Tamnes, C. K., Nygaard, E., Bjørk, R. F., Stavrinou, M., & Espeseth, T. (2021). A pilot study of a parent emotion socialization intervention: Impact on parent behavior, child self-regulation, and adjustment. *Frontiers in psychology*, 4552. <https://doi.org/10.3389/fpsyg.2021.730278>

Chan, R. F. Y., Qiu, C., & Shum, K. K. M. (2021). Tuning in to kids: A randomized controlled trial of an emotion coaching parenting program for Chinese parents in Hong Kong. Developmental Psychology, 57(11), 1796. <https://doi.org/10.1037/dev0001258>

Edrissi, F., Havighurst, S. S., Aghebati, A., Habibi, M., & Arani, A. M. (2019). A pilot study of the tuning in to kids parenting program in Iran for reducing preschool children’s anxiety. *Journal of Child and Family Studies*, 28(6), 1695-1702. <https://doi.org/10.1007/s10826-019-01400-0>

Foroughe, M., Stillar, A., Goldstein, L., Dolhanty, J., Goodcase, E. T., & Lafrance, A. (2019). Brief emotion focused family therapy: An intervention for parents of children and adolescents with mental health issues. *Journal of marital and family therapy*, 45(3), 410-430. <https://doi.org/10.1111/jmft.12351>

Havighurst, S. S., Harley, A., & Prior, M. (2004). Building preschool children's emotional competence: A parenting program. *Early Education & Development*, *15*(4), 423-448. <https://doi.org/10.1207/s15566935eed1504_5>

Havighurst, S. S., Wilson, K. R., Harley, A. E., Kehoe, C., Efron, D., & Prior, M. R. (2013). “Tuning into kids”: Reducing young children’s behavior problems using an emotion coaching parenting program. *Child Psychiatry & Human Development*, 44(2), 247-264. <https://doi.org/10.1007/s10578-012-0322-1>

Havighurst, S. S., Kehoe, C. E., & Harley, A. E. (2015). Tuning in to teens: Improving parental responses to anger and reducing youth externalizing behavior problems. *Journal of adolescence*, *42*, 148-158. <https://doi.org/10.1016/j.adolescence.2015.04.005>

Havighurst, S. S., Kehoe, C. E., Harley, A. E., Radovini, A., & Thomas, R. (2022). A randomized controlled trial of an emotion socialization parenting program and its impact on parenting, children's behavior and parent and child stress cortisol: Tuning in to Toddlers. *Behaviour Research and Therapy*, 149, 104016. <https://doi.org/10.1016/j.brat.2021.104016>

Havighurst, S. S., Murphy, J. L., & Kehoe, C. E. (2021). Trauma-Focused Tuning in to Kids: Evaluation in a Clinical Service. *Children*, 8(11), 1038. <https://doi.org/10.3390/children8111038>

Havighurst, S. S., Wilson, K. R., Harley, A. E., & Kehoe, C. E. (2019). Dads Tuning in to Kids: A randomized controlled trial of an emotion socialization parenting program for fathers. *Social Development*, 28(4), 979-997. <https://doi.org/10.1111/sode.12375>

Havighurst, S. S., Wilson, K. R., Harley, A. E., & Prior, M. R. (2009). Tuning in to kids: an emotion‐focused parenting program—initial findings from a community trial. *Journal of community psychology*, *37*(8), 1008-1023. <https://doi.org/10.1002/jcop.20345>

Havighurst, S. S., Wilson, K. R., Harley, A. E., Prior, M. R., & Kehoe, C. (2010). Tuning in to Kids: improving emotion socialization practices in parents of preschool children–findings from a community trial. *Journal of Child Psychology and Psychiatry*, *51*(12), 1342-1350. <https://doi.org/10.1111/j.1469-7610.2010.02303.x>

Kehoe, C. E., Havighurst, S. S., & Harley, A. E. (2014). Tuning in to teens: Improving parent emotion socialization to reduce youth internalizing difficulties. *Social Development*, *23*(2), 413-431. <https://doi.org/10.1111/sode.12060>

Kehoe, C. E., Havighurst, S. S., & Harley, A. E. (2015). Somatic complaints in early adolescence: The role of parents’ emotion socialization. *The Journal of Early Adolescence*, *35*(7), 966-989. <https://doi.org/10.1177/0272431614547052>

Kehoe, C. E., Havighurst, S. S., & Harley, A. E. (2020). Tuning in to Teens: Investigating moderators of program effects and mechanisms of change of an emotion focused group parenting program. *Developmental Psychology*, *56*(3), 623. <https://doi.org/10.1037/dev0000875>

Lambie, J. A., Lambie, H. J., & Sadek, S. (2020). “My child will actually say ‘I am upset’… Before all they would do was scream”: Teaching parents emotion validation in a social care setting. *Child: Care, Health and Development,* 46(5), 627-636. <https://doi.org/10.1111/cch.12770>

Lauw, M. S., Havighurst, S. S., Wilson, K. R., Harley, A. E., & Northam, E. A. (2014). Improving parenting of toddlers’emotions using an emotion coaching parenting program: a pilot study of tuning in to toddlers. *Journal of Community Psychology*, 42(2), 169-175. <https://doi.org/10.1002/jcop.21602>

Leung, C., Chan, S., Ip, H. L., Szeto, H., Lee, M., Chan, K., & Chan, M. (2020). Effectiveness of parenting program for Macau shift work parents: Randomized controlled trial. Research on Social Work Practice, 30(6), 612-622. <https://doi.org/10.1177/1049731520903429>

Mastromanno, B. K., Kehoe, C. E., Wood, C. E., & Havighurst, S. S. (2021a). A randomised-controlled pilot study of the one-to-one delivery of Tuning in to Kids: impact on emotion socialisation, reflective functioning, and childhood behavior problems. *Emotional and Behavioral Difficulties*, *26*(4), 359-374. <https://doi.org/10.1080/13632752.2021.1984208>

Mastromanno, B. K., Kehoe, C. E., Wood, C. E., & Havighurst, S. S. (2021b). Tuning in to Kids: Clinical Case Studies from One-to-One Delivery. *Clinical Case Studies*, *20*(4), 267-282. <https://doi.org/10.1177/153465012098390>

Pezeshki, P., Doos Ali Vand, H., Aslzaker, M., & Jafari, M. (2020). The Effectiveness of Emotion Coaching Parenting Program in Iranian Preschool Children With Internalizing Disorders. <https://doi.org/10.32598/jpcp.8.3.676.2>

Qiu, C., & Shum, K. K. M. (2022). Emotion coaching intervention for Chinese mothers of preschoolers: A randomized controlled trial. Child Psychiatry & Human Development, 53(1), 61-75. <https://doi.org/10.1007/s10578-020-01101-6>

Rolock, N., Ocasio, K., White, K., Havighurst, S., Cho, Y., Fong, R., ... & Faulkner, M. (2021). Tuning in to Teens (TINT) with adoptive parents and guardians in the US: the replication phase of intervention research. Journal of public child welfare, 15(1), 22-51. <https://doi.org/10.1080/15548732.2020.1846660>

Shortt, J. W., Eddy, J. M., Sheeber, L., & Davis, B. (2014). Project home: a pilot evaluation of an emotion-focused intervention for mothers reuniting with children after prison. Psychological services, 11(1), 1. <https://doi.org/10.1037/a0034323>

Wilhelmsen-Langeland, A., Aardal, H., Hjelmseth, V., Fyhn, K. H., & Stige, S. H. (2020). An emotion focused family therapy workshop for parents with children 6-12 years increased parental self-efficacy. *Emotional and Behavioural Difficulties,* 25(1), 29-41. <https://doi.org/10.1080/13632752.2019.1655921>

Wilson, K. R., Havighurst, S. S., & Harley, A. E. (2012). Tuning in to Kids: an effectiveness trial of a parenting program targeting emotion socialization of preschoolers*. Journal of family Psychology*, 26(1), 56. <https://doi.org/10.1037/a0026480>

Wilson, K. R., Havighurst, S. S., & Harley, A. E. (2014). Dads tuning in to kids: piloting a new parenting program targeting fathers’emotion coaching skills. *Journal of Community Psychology,* 42(2), 162-168. <https://doi.org/10.1002/jcop.21601>

Wilson, K. R., Havighurst, S. S., Kehoe, C., & Harley, A. E. (2016). Dads tuning in to kids: Preliminary evaluation of a fathers' parenting program. *Family Relations*, 65(4), 535-549. <https://doi.org/10.1111/fare.12216>
